# Caecal volvulus in a 35-year-old man: a case report

**DOI:** 10.11604/pamj.2023.44.37.37403

**Published:** 2023-01-19

**Authors:** Abdulrahman Tawfeeq Abdulmuttaleb, Zakareya Al-Habbal, Faisal Ahmed

**Affiliations:** 1 Department of Surgery, Al-Thawra Private Hospital, Taiz, Yemen,; 2 Department of Surgery, School of Medicine, University of Kurdistan, Erbil, Iraq,; 3 Department of Urology, School of Medicine, Ibb University of Medical Sciences, Ibb, Yemen

**Keywords:** Intestinal obstruction, adult, colicky abdominal pain, caecal volvulus, case report

## Abstract

Caecal volvulus is a rare cause of mechanical bowel obstruction (1-1.5%) that carries a high mortality rate if diagnosis or surgical intervention is delayed. We report a 35-year-old man who presented with acute colicky abdominal pain, vomiting, and constipation for the past 18 hours. A plain abdominal X-ray showed distended large bowel loops with two large well-defined air-fluid levels superimposed on each other, suggestive of caecal volvulus. The patient underwent emergency laparotomy, and the intraoperative finding confirmed the diagnosis of gangrenous extended caecal volvulus, which involves the terminal ileum, cecum, and the whole of the ascending colon. A right hemicolectomy was performed, and bowel continuity was restored by primary ileotransverse anastomosis. The patient recovered without complications and was discharged on postoperative day 6. In conclusion, caecal volvulus is a rare cause of adult intestinal obstruction. Early diagnosis and surgical intervention can prevent perforation and reduce morbidity related to volvulus perforation.

## Introduction

Colonic volvulus, which was formerly termed intestinal strangulation, was first described by Rokitansky in 1873 [1]. Organoaxial or cecocolic volvulus is a cecal volvulus subtype where the ascending colon and distal ileum twist clockwise around each other [2]. Caecal volvulus is a rare cause of mechanical intestinal obstruction, accounting for 1-1.5% of all intestinal obstructions and approximately 11% of all volvulus-related bowel obstructions [3]. The prognosis of the disease may be poor depending on bowel viability, the time elapsed between the onset of symptoms, diagnosis and intervention, the presence of a comorbid condition, and the patient's age [3]. There are sparse reports of caecal volvulus in adults in the literature [1,4,5]. Here we report a 35-year-old male patient who presented with acute abdominal pain and was radiologically and intraoperatively diagnosed as caecal volvulus.

## Patient and observation

**Patient information:** a 35-year-old male patient presented to our emergency department in Al-Thawra Hospital, Taiz, Yemen, in January 2021, complaining of acute severe diffuse colicky abdominal pain, vomiting, and constipation for the past 18 hours. The vomiting was a green color and had bilious intestinal content. Otherwise, he had no prior history of such problems, weight loss, or rectal bleeding. The patient is a Khat chewer with a history of chronic constipation. The patient mentioned a history of appendectomy ten months ago. There was no history of a genetic disorder, family history of bowel disease, or malignancy.

**Clinical findings:** in the initial evaluation, the patient was pale, looked ill, febrile with an oral temperature of 38°C, tachypneic with a pulse rate of 120 beats per minute, and blood pressure of 90/60 mm Hg; he was in impending shock. The patient’s abdomen was moderately distended, mainly at the left upper quadrant (LUQ), with tenderness and rebound tenderness, and bowel sounds were hypokinetic. Timeline of current episode: following 18 hours of starting abdominal pain, the patient was hospitalized and blood and radiologic investigations were performed, and urgently transferred to the operating room.

**Figure 1:**
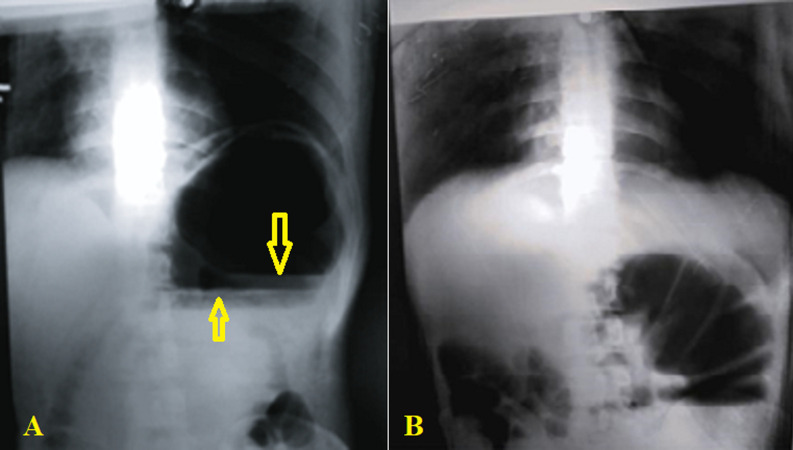
plain abdominal radiography showing two large well-defined air-fluid levels approximately 15 cm in width superimposed on each other located at the left upper quadrant of the abdomen (arrows), with minimal gas seen at the distal bowel loops; A) erect view; B) supine view

**Diagnostic assessment:** blood examination showed white blood cell (WBC) 18.3 x 10^3^/ml with 90% polymorph neutrophils (leukocytosis with neutrophilic predominance), hemoglobin: 14 g/dL, and platelets count: 200 x 10^3^/mcL. Other blood investigation tests, such as liver function tests, coagulation tests, and renal function tests, were within normal limits. The patient's EKG was normal except for tachycardia. Erect and supine abdominal radiography X-rays showed two large well-defined air-fluid levels superimposed on each other located at the LUQ of the abdomen, with minimal gas seen at the distal bowel loops, suggestive of caecal volvulus (Figure 1 (A, B)). Computed tomography (CT) scan is unavailable in our hospital and was not performed.

**Diagnosis:** a provisional diagnosis was made depending on history, physical examination, and radiography X-ray findings.

**Figure 2:**
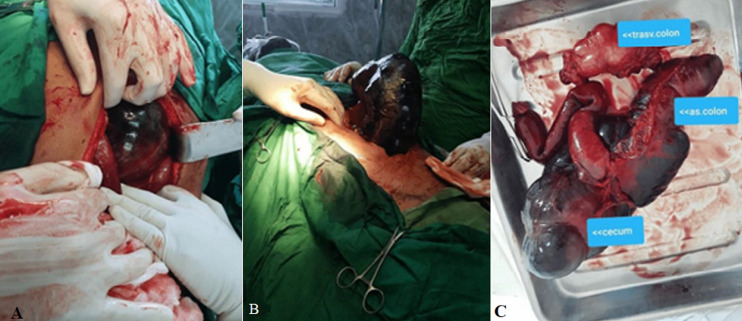
intraoperative photos showing; A) darkly colored cecum with previous appendectomy scar; B) gangrenous caecal volvulus; C) the excised terminal ileum, cecum, and whole ascending colon

**Therapeutic interventions:** after quick resuscitation with intravenous fluids (one-liter normal saline), intravenous antibiotic (ceftazidime and metronidazole), and nasogastric suction, an urgent laparotomy was performed through a midline incision. Intraoperative findings confirmed the presence of a large cecocolic volvulus; involving the terminal ileum, cecum, and the whole of the ascending colon. There was a long cecal mesentery (floating cecum) intermingled with the adhesion band from the recent appendectomy. The cecum was hugely distended, located at the left hypochondrium, pushing the empty stomach totally to the right of midline. All the involved bowels were ischemic and gangrenous except the hepatic flexure (Figure 2(A, B, C)). A right hemicolectomy was performed, and bowel continuity was restored by primary ileotransverse anastomosis. The small bowel and cecum were laden with a thick greenish content from daily chewing of a plant called Qat or Khat, which can sometimes cause chronic constipation. The total operative time was 200 minutes, and the whole blood loss was 300 mL.

**Follow-up and outcome:** the patient had an adequate postoperative evolution, decreased leukocyte count, and maintained antibiotics with ceftazidime (2 g IV q8hr for four days) and metronidazole (500 mg IV q8hr for four days). After confirming adequate bowel peristalsis, a liquid diet was started on the second postoperative day. On the third postoperative day, a soft diet was started and tolerated. The patient was discharged from the hospital on the 6^th^ postoperative day. The histopathology report showed necrotic bowel, evidence of ischemic changes, and focal ulcerations with ileitis, typhlitis, and colitis.

**Patient perspective:** the patient was pleased with the care he received throughout therapy.

**Informed consent:** written informed consent was obtained from the patient for participation in our study.

## Discussion

This paper describes a successful surgical intervention of a rare case of caecal volvulus in a 35-year-old adult patient. Caecal volvulus is a rare cause of mechanical intestinal obstruction [5]. Its incidence ranges from 2.8-7.1 per million people per year, with female predominance (1: 3.7), and it is responsible for 1-1.5% of all intestinal obstruction and 11% of all volvulus-related intestinal obstruction, with a high mortality rate of up to 40% [3]. Patients aged between 40 and 62 years are commonly affected by caecal volvulus [4]. In the present case, the patient was male and aged 35 years. Our findings contributed to the exciting outliers in the demographic characteristics for caecal volvulus. Caecal volvulus can be organoaxial (cecocolic or true cecal volvulus); in which ascending colon and distal ileum twist clockwise around each other, as seen in our case or mesentericoaxial (cecal bascule), in which the caecum, however, is not totally fixed and positioned anteriorly over the ascending colon at a right angle to the mesentery [2].

The most prevalent type of volvulus is sigmoid volvulus accounting for 60%, followed by caecal volvulus accounting for 11%. Few reports of volvulus involving the transverse colon, including the splenic or hepatic angle [4]. The reported caecal volvulus predisposing factors are colonic mass, laxative abuse, adequate caecal movement, bascule formation, adhesions from previous abdominal surgery, chronic constipation, pregnancy, prolonged immobility, patients with mental disorders, Chagas disease, and pregnancy, as more than 10% of caecal volvulus cases involve pregnant women [4-6]. In our case, an adhesion band from a recent appendectomy and chronic constipation, most likely from a habit of Qat chewing, were predisposing factors for caecal volvulus [7]. Laboratory tests are neither specific nor sensitive for detecting caecal volvulus. However, they can indicate the degree of obstruction and severity of the problem [4]. Our patient had a high leukocytosis level with an elevated neutrophil count.

Abdominal radiography X-ray makes a diagnosis in 60 to 70% of cases, demonstrating four typical signs: caecal dilation, presence of air-fluid level on the right side of the abdomen (in our case, it was seen in LUQ with two air-fluid levels), a minimal amount of gas in the distal colon, and the “coffee bean sign” which may be present in 50% of cases [2,8]. A CT scan is the cornerstone and key to a dependable diagnosis. It is the most useful and reliable single radiological procedure with accurate diagnosis in more than 90% of cases. It can determine a classical distended bowel segment, twists at the mesenteric base, twisted blood vessels, and changes in bowel walls, and it may show one or more of the following signs: “whirl signs”, “bird beak”, “coffee bean”, and “split wall signs” [8,9]. In our case, the CT scan was unavailable in our city, and the patient's condition worsened, limiting us from transferring them to another center.

It is generally agreed that early surgical intervention is the only effective treatment for caecal volvulus. Many surgical procedures can be used in caecal volvulus management, such as laparoscopic surgery (best choice) and open surgery, which is the second-best option in the form of right hemicolectomy and primary ileo-transverse anastomosis. It is associated with good results in more than 70% of cases [3,10]. Untwisting with or without cecopexy is not recommended as it is associated with 60-70% recurrence [3,11]. Colonoscopic detorsion is not recommended due to the high incidence of perforation and recurrence [6]. After exploration, if the intraoperative finding is caecal volvulus, the bowel viability should be evaluated. If gangrenous or perforated intestines are found, the non-viable intestines should be removed. Right hemicolectomy with primary ileo-transverse anastomosis is the treatment of choice for all gangrenous or perforated bowels. An ileostomy may be recommended depending on the patient's intraoperative findings and condition [12]. Our patient had minimally contaminated caecal volvulus, and we performed a right hemicolectomy with primary ileotransverse anastomosis.

## Conclusion

Caecal volvulus is a rare fatal cause of adult intestinal obstruction. Physical examination and radiological results should yield a diagnosis as soon as possible. Treatment consists of immediate surgical exploration to reduce the mortality rate, as performed in our case.
